# Association between Interactions among ACE Gene Polymorphisms and Essential Hypertension in Patients in the Hefei Region, Anhui, China

**DOI:** 10.1155/2023/1159973

**Published:** 2023-04-13

**Authors:** Li Wang, Ting-ting Song, Chang-wu Dong

**Affiliations:** ^1^College of Traditional Chinese Medicine, Anhui University of Traditional Chinese Medicine, Anhui Hefei 230012, China; ^2^The Second Clinical Medical School, Anhui University of Traditional Chinese Medicine, Anhui Hefei 230061, China

## Abstract

**Objective:**

Essential hypertension (EH) is a common cardiovascular disease that endangers human health. Its pathogenesis is complex and has not been fully elucidated. We explore the association between EH and interactions among polymorphisms of the angiotensin converting enzyme (ACE) gene in the Hefei region, Anhui, China.

**Methods:**

A total of 500 participants (400 hypertensive and 100 normotensive) were included in this study. The polymorphisms were detected via improved multiple ligase detection reaction (iMLDR). To improve the accuracy of prediction, multifactor dimensionality reduction (MDR) was used to analyze the overall effect of interactions among seven loci on the incidence of EH.

**Results:**

The frequencies of polymorphisms in the ACE genes rs12709426, rs4291, rs4309, rs4331, rs4343, rs4459609, and rs4461142 in the EH group were not statistically significantly different from those in the control group. We also found that the single nucleotide polymorphism (SNP) rs12709426 only had a homozygous AA genotype and no polymorphisms. There were no differences in the frequency of genetic polymorphisms between the EH and control groups. The best model explaining the EH group was the combined effect of ACE genes rs4291, rs4309, and rs4461142.

**Conclusion:**

There is an interaction effect among ACE gene loci in EH patients in Hefei region, Anhui, China. Also, the ACE gene SNP rs12709426 only has a homozygous AA genotype and does not show an association with EH.

## 1. Introduction

Essential hypertension (EH) is a common disorder associated with increased cardiovascular morbidity and mortality [1]. It is a major risk factor for coronary heart disease, cerebral infarction, and other cardiovascular and cerebrovascular diseases. Every year, approximately 7.5 million people die of EH worldwide [2]. It is a polygenic hereditary disease that is affected by both genes and environmental factors. Gene-gene and/or gene-environment interactions play an important role in its occurrence and development [3]. EH is a complex, polygenic condition with no single causative agent. Despite advances in our understanding of its pathophysiology, hypertension remains one of the world's leading public health problems. Indeed, risk for EH seems to depend on a complex network of interactions among genes and environmental factors [4].

Some SNPs have been found to be closely associated with blood pressure. Among more than 150 genes related to the pathogenesis of EH, those related to the renin-angiotensin system (RAS) play a crucial role in regulating blood pressure; the RAS can affect vascular volume, cardiovascular remodeling, and sodium and water balance in the body in a variety of ways. The dysfunction of, or quantitative variation in, RAS components can have a profound impact on the pathogenesis of hypertension. As an essential component of RAS, genetic variation in ACE genes plays a pivotal role in the formation of hypertension. Also known as kininase II, ACE is a class of peptidase. It cleaves Ang I to form Ang II. Ang II, the main active peptide of the RAS, causes the contraction of smooth muscle cells along the blood vessel wall and the secretion of aldosterone from zona glomerulosa cells of adrenal gland, increasing the reabsorption of sodium in the kidney [5, 6]. These mechanisms serve as the key factors leading to cardiovascular diseases, hypertension in particular [7]. In recent years, studies have investigated the relationship between ACE genes on both national and regional scales. Variation in SNP loci among different regions, races, and individuals, leading to different degrees of susceptibility to EH, has been reported [8]. Considering the large number and wide distribution of SNPs, the accumulation of multiple SNP loci or specific combinations has an effect on disease susceptibility. Indeed, multiple genes may be associated with EH, and its regulatory mechanism is very complex. Its pathogenesis is likely caused by several genes and loci. Thus, it is important to study the potential interactions among genes and loci and understand the overall association on EH. To this end, in this study, we investigated the potential interactions among seven different loci of the ACE gene (rs12709426, rs4291, rs4309, rs4331, rs4343, rs4459609, and rs446112).

## 2. Materials and Methods

### 2.1. Study Participants

In all, 400 Chinese Han patients with hypertension were selected as study participants. Among them, 192 were male and 208 were female. The average age was 67.97 ± 12.88 years old. The control group was composed of 100 healthy individuals, with a mean age of 63.76 ± 11.37 years old. All selected cases were recruited from the Department of Cardiovascular Internal Medicine, the First Affiliated Hospital of Anhui University of Chinese Medicine, Anhui, China, from March 2022 to May 2022.

### 2.2. Study Selection Criteria

Included studies met the following criteria: (1) age greater than 18 years old, male or female; three daily measurements of systolic blood pressure (SBP) ≥ 140 mmHg or diastolic blood pressure (DBP) ≥ 90 mmHg while sitting, following the diagnostic criteria stipulated in the 1999 WHO/ISH Hypertension Treatment Guidelines [9]; (2) free of *β*-blockers, angiotensin converting enzyme inhibitors (ACEIs), and angiotensin receptor blockers (AT1Rs) before drawing blood. If the aforementioned drugs were taken, substitute *α*-blockers were prescribed instead for a period of five half-lives after inclusion; and (3) written informed consent was obtained from each participant.

Studies that met the following criteria were excluded: (1) participants with secondary hypertension or malignant hypertension; (2) severe valvular heart disease, cardiomyopathy, or unstable angina within 6 months; (3) severe kidney and liver dysfunction; (4) pregnant and lactating women; (5) alcoholism, drug abuse, or with a history of mental illness; (6) participants with malignant tumors or diseases associated with vital systems; and (7) disability or with foreseeable reasons to prevent follow-ups and noncooperators.

The participants in the control group were diagnosed as nonhypertensive according to the same diagnostic criteria; other inclusion and exclusion criteria were the same for both the patient and the control groups. Two investigators independently determined whether the studies met the inclusion criteria, and a third investigator was available for resolving any disputes.

### 2.3. Data Collection

The following data were collected from the patient and control groups: age, gender, smoking, alcohol consumption, salt intake, family history, SBP, DBP, body mass index (BMI), and levels of glucose (GLU), serum total cholesterol (TC), serum triglycerides (TG), high-density lipoprotein cholesterol (HDL-C), and low-density lipoprotein cholesterol (LDL-C). Smoking was defined as positive when a participant had smoked at least one cigarette per day in the past 6 months. Alcohol consumption was defined as positive when a participant had consumed hard liquor, beer, or wine at least one time per week in the past 6 months. Salt intake was divided into the following two categories: >6 g and <6 g. Family history of hypertension was considered positive when a participant had at least one parent, sibling, or offspring diagnosed with hypertension. Height and weight were measured twice, and the average was used for further analyses. BMI was calculated by dividing the weight (kg) by the square of the height (m). SBP and DBP were measured twice (right arm), using a standardized mercury sphygmomanometer after the participant rested for 30 min in a seated posture; average SBP and DBP values were used for further analyses.

### 2.4. Methods

(1) DNA extraction: take 5 mL fasting blood samples from the median cubital vein after routine disinfection, using EDTA anticoagulant, and store them in a -20°C refrigerator. Extract DNA within a week and store at -80°C. (2) PCR and SNP sequencing methods: the PCR primers are given in Table 1. In all, 500 samples were analyzed for SNP sites using an iMLDR multiple SNP genotyping kit (Shanghai Tianhao Biotechnology Co., Ltd., China). The reaction system (10 *μ*L) included 1x GC-I buffer (Takara), 3.0 mM Mg2+, 0.3 mM dNTP, 1 U HotStarTaq polymerase (Qiagen Inc.), 1 *μ*L sample DNA, and 1 *μ*L multiplex PCR primer. (3) SNP sequencing: the connection products were fed into an ABI3730XL sequencer. The data were analyzed using GeneMapper 4.1 (Applied Biosystems, USA)

### 2.5. Statistical Analysis

The data collected were tabulated in Excel and then analyzed with SPSS 23.0. Continuous variables are presented as means with standard deviations. Categorical variables are presented as the frequencies or the percentages. For categorical variables, weights were calculated to eliminate any possible bias. The *T* test or chi-square test was used to detect differences among participant characteristics. *P* value ≤ 0.05 was considered statistically significant. OR values and 95% confidence intervals were derived. The Hardy-Weinberg equilibrium law was employed to test genetic equilibrium. The frequencies of alleles were also calculated. Multifactor dimensionality reduction (MDR) (version 2.0) was used to analyze the overall effect of interactions among seven loci on the incidence of EH.

## 3. Results

### 3.1. Characteristics of the Study Population

The demographic and clinical characteristics of the study participants are presented in Table 2. There were significant differences in alcohol consumption, salt intake, family history, BMI, and levels of GLU, LDL-C, and HDL-C between the EH and control groups.

### 3.2. Association between ACE Gene Polymorphisms and EH

#### 3.2.1. Hardy-Weinberg Equilibrium Test

The frequencies of the ACE gene loci rs12709426, rs4291, rs4309, rs4331, rs4343, rs4459609, and rs446112 complied with the Hardy-Weinberg equilibrium with corresponding quality to serve as representatives of a population. The details are shown in Table 3.

#### 3.2.2. Polymorphisms of the ACE Gene

The frequencies of polymorphisms in the ACE genes rs12709426, rs4291, rs4309, rs4331, rs4343, rs4459609, and rs446112 in the EH group were not statistically significantly different from those in the control group. In addition, we found that rs12709426 only has a homozygous AA genotype, being void of heterozygotes, variation, and gene polymorphisms. There were no differences in the frequency of genetic polymorphisms between the EH and control groups. This demonstrates that rs12709426 has no association with EH. The details are shown in Table 4.

#### 3.2.3. Effect of Interactions among ACE Gene Loci on the Pathogenesis of EH

In this study, seven loci of the ACE gene were included for MDR analysis of interactions among sites. The best model was determined by calculating the consistency of cross-validation, the training and testing balanced accuracies, and the combination of the maximum consistency of the cross-validation and the maximum test accuracy (0.6112). The best model explaining the EH group was the combined effect of ACE genes rs4291, rs4309, and rs4461142. The details are shown in Table 5.

## 4. Discussion

SNP is a powerful tool for the discovery of high-risk populations and the identification of disease-related genes. According to data from the Human Genome Project, gene loci can vary by region and race, which leads to different susceptibilities to EH. There are still many challenges to resolve to understand the pathogenesis of EH. In recent years, much research has been carried out on ACE, the key gene involved in the RAS system, but most studies focus on a single site. Previous studies of ACE gene polymorphisms and EH have reported mixed results [10]. Since I/D in the 16 introns of the ACE gene has been detected, numerous studies have been performed for ACE gene polymorphism. However, there is still a dearth of clear results. A meta-analysis of ACE I/D polymorphism and EH in the Chinese population reported controversial results, noting that the D allele was associated with EH in the Han, Kazakh, Tibetan, and Zhuang groups, but in the national minorities of Mongolian, Uighur, Yi, Dongxiang, Yugur, Korean, and Gamel, no association was found [11, 12]. In other regions, studies have also found that the DD genotype and the D allele were significantly associated with hypertension in Egyptian patients and Saudi patients [13, 14], but ACE I/D polymorphism was not a significant factor for hypertension in the Tunisian or South Sulawesi Indonesian populations [15, 16]. As for A2350G, Niu et al. [17] revealed that the A allele was associated with an elevated risk of EH in the Chinese Han group. Yang et al. [18] discovered that the A allele could increase the risk of EH in the Chinese Yi ethnic group, whereas Pan et al. [19] noted that A2350G polymorphism was associated with left ventricular hypertrophy, not EH, in southern Han Chinese. There are other sites that have been studied, such as the ACE rs4291, rs4335, and rs4363 polymorphisms, which may not be associated with hypertension but may trigger early signal metabolic changes in the hypertensive process [20]. SNPs of ACE rs4318, rs4343, rs4344, rs4362, and rs4353 have been reported to be associated with an increased risk of developing high blood pressure, coronary heart disease, and myocardial infarction [21–23]. In young people with hypertension, there is linkage disequilibrium between the rs12709426 site of the ACE gene and the single nucleotide polymorphism of the ACE gene promoter, with the former being associated with EH incidence [24]. However, there has been no case report of the homozygous ACE gene rs12709426. In this study, we found that the ACE gene SNP rs12709426 has only a homozygous AA genotype in EH patients in Hefei, Anhui Province, China, with no variation and no genetic site polymorphism. There were no differences in the genotype polymorphism frequency between the EH group and the control group, and the SNP was not significantly correlated with EH. Our results help elucidate the pathogenesis of EH and provide meaningful guidance for further study of the differences in susceptibility and pathogenesis of EH across different regions.

The above studies show that the variations in gene-disease associations are due to genetic heterogeneity, population stratification and sampling bias, differences in the complex genetic background, environmental complications, disparity in model assumptions, and gene-gene interactions [25]. The interactions among genes and/or loci have rarely been investigated. Previous studies have found potential interactions between ACE, AGT, angiotensin II type 1 receptor (AGTR1), and *α* adducin (ADD1) variants and correlations between them and clinical endpoints in individuals with hypertension [26]. In this study, which used generalized multifactor dimensionality reduction (GMDR) analysis to identify models describing gene interactions, the best disease-conferring model between genes was the 5-locus model comprising the variants AGT −217G/A, −20A/C, −6G/A, 235M/T, and ACE I/D. And gene-gene interactions were reliable and promising markers for predisposing one to hypertension. Interactions among genetic loci may be used to predict susceptibility to hypertension. Additionally, some scholars use logistic regression to analyze the interaction between genes, and the results showed that ACE I/D and CYP11B2-344 T/C polymorphisms may interact, based on the multiplicative model [27]. The risk of having EH for ACE I/D DD and CYP11B2-344 T/C TC genotype carriers was 3.04 times that for the ACE I/D II genotype and CYP11B2-344 T/C TT genotype carriers (95% CI 1.25–7.39). The other genotypes did not show any interactions in this study, based on the multiplicative model. Niu et al. [28] also found a strong synergistic effect between ACE insertions/deletions and CY11B2 T-344C, wherein the combination increases susceptibility to EH. Furthermore, there were significant interactions between the genetic and environmental factors, which likely increase the risk of suffering from EH. Another study analyzed the superposition effect and interactions among six loci: AGT M235T, ACE I/D, eNOS Glu298ASP, ET-2 A985G, ANP T2238C, and NPRC A-55C [29]. In the patient group, the frequencies of five genetic polymorphisms—AGT M235 (MM, MT, TT), ACE I/D (II, ID, DD), eNOS Glu298ASP, ET-2 A985G (AA, AG, GG), and ANP T2238C (TT, TC, CC)—showed no significant difference compared to those of the control group. However, in the patient group, the CC genotype frequency (0.540) and the C allele genotype frequency (0.770) of NPRC A-55C were significantly different from those of the control group. In the analysis of gene-gene interactions using the MDR method, although ACE I/D on its own was not the best model, it was nonetheless included in the optimal ACE/eNOS/NPRC 3-locus interactive model and in the ACE/eNOS/ET-2/NPRC 4-locus interactive model. The results indicated that the ACE insertion/deletion polymorphism is not a major EH gene but may have a synergistic effect with other genes.

The above-mentioned results further emphasize the effects of genetic heterogeneity and multiple gene-gene interactions in the etiology of EH. We investigated the potential interactions among seven different loci of the ACE gene. Our study exposed the interaction among seven different loci of the ACE gene as a potential disease modifier to influence the biological and biochemical pathways underlying the disease pathophysiology compared to individual loci of genes. Due to the high heterogeneity of EH, its long disease course, and its slow progression, different mechanisms are involved in different stages, and an abnormality in or absence of one mechanism is often compensated for by other mechanisms [30, 31]. In the present analysis, we found that the results varied in many studies, which confirms that the action mode of minor genes plays an important role in EH genetic mechanisms. Furthermore, there is accumulation between loci, and when the threshold of disease occurs, microeffect genes are involved in the genetic susceptibility of EH. Although the number of such genetic variants is large, the effect of each is relatively weak. The influence of a single gene variation is not enough to cause EH, and a combination of multiple genes is needed to cause an individual to develop the disease.

## 5. Conclusions

In summary, this study demonstrated that there is an interaction effect among ACE gene loci in EH patients in Hefei region, Anhui, China. Also, the ACE gene SNP rs12709426 only has a homozygous AA genotype and does not show an association with EH. Furthermore, the in-depth analysis of gene-gene and gene-environment interactions plays an important role in exploring the etiology of EH and lays a foundation for the screening of hypertension-prone patients, subsequent prevention and treatment, and drug research and development. In addition, regional and ethnic differences should be fully taken into account when using gene polymorphisms to guide decision-making regarding hypertension. With the deepening of research on gene polymorphisms, the genetic mechanisms underlying EH will gradually be identified, and truly individualized medicine will be achieved.

## Figures and Tables

**Table 1:** Oligonucleotide primers used for the multiplex PCR amplification of the ACE gene.

SNP	5′-3′	SNP sequencing
rs4459609F	CAGCGTTGCCATGAAGATGAAAT	A5466C
rs4459609R	AAGGACGTGTGGTGGGGAAGTT	
rs12709426F	CTGTGCCCATGGTACCCACTCT	A3892G
rs12709426R	CACTGGGTGACTGGCTGGAAGT	
rs4291F	CCCCGGCCTTGTCACTCC	A240T
rs4291R	GAAGCTGGAGAAAGGGCCTCCT	
rs4309F	ATGGACCAGCTCTCCACAGTGC	T1237C
rs4309R	CCCATACCCGTGTCATTGGTGA	
rs4331F	CACCCAGGCCAGGAAGTTTGAT	G2215A
rs4331R	AGACCCTGTGTTGGGCTCACTG	
rs4343F	CCCCTACCAGATCTGACGAATGTG	G2350A
rs4343R	CCTAGGCTTGGGGTTTCACAGC	
rs4461142F	CAGCGTTGCCATGAAGATGAAAT	I/D
rs4461142R	AAGGACGTGTGGTGGGGAAGTT	

**Table 2:** Comparison of general clinical data between the EH and control groups.

Characteristic	Group	EH group	Control group	*x* ^2^/*t*	*P*	OR (95% CI)
		*N* = 400	*N* = 100			
Age (years)		67.97 ± 12.88	63.76 ± 11.37	0.325	0.783	1.03 (0.65-1.78)
Gender	Male	192	44	0.51	0.474	1.17 (0.76-1.83)
	Female	208	56			
Smoking	Yes	129	33	0.02	0.886	0.96 (0.61-1.54)
	None	271	67			
Alcohol consumption	Yes	82	9	7.11	0.008^∗^	2.61 (1.26-5.39)
	None	318	91			
Salt intake	≤6 g/day	189	64	8.98	0.003^∗^	1.99 (1.26-3.12)
	>6 g/day	211	36			
Family history	Yes	179	22	17.22	<0.001^∗^	
	None	221	78			
SBP (mmHg)		138.58 ± 15.87	117.42 ± 13.68	12.244	<0.001^∗^	
DBP (mmHg)		82.16 ± 9.83	75.36 ± 8.14	6.383	<0.001^∗^	
BMI		21.58 ± 2.773	20.48 ± 3.271	-3.410	0.001^∗^	
GLU (mmHg)		5.67 ± 1.70	5.05 ± 0.65	5.783	<0.001^∗^	
TC (mmHg)		4.32 ± 1.39	4.38 ± 0.84	-0.544	0.587	
TG (mmHg)		3.28 ± 18.69	1.03 ± 0.46	1.202	0.498	
LDL-C (mmol/L)		2.62 ± 1.32	1.65 ± 0.27	13.518	<0.001^∗^	
HDL-C (mmol/L)		1.25 ± 0.47	1.60 ± 0.37	-6.939	<0.001^∗^	

^∗^ *P* < 0.05.

**Table 3:** The ACE gene-locus Hardy-Weinberg equilibrium test table.

SNP	Alleles	MAF (this experiment)		MAF (HapMap-HCB)		Call rate (%)	Test for HWE (*P* value)
rs12709426 (A3892G)	A/G	G	0	G	0.004	100	1
rs4291 (A240T)	A/T	T	0.349	T	0.32	100	0.0756
rs4309 (T1237C)	T/C	C	0.36	C	0.281	98.4	0.0853
rs4331 (G2215A)	G/A	A	0.326	A	0.281	100	0.0118
rs4343 (G2350A)	G/A	G	0.325	G	0.274	100	0.0141
rs4459609 (A5466C)	A/C	C	0.354	C	0.303	100	0.0861
rs4461142 (I/D)	C/T	T	0.432	T	0.358	98.8	0.145

**Table 4:** Comparison of ACE genotypes between the EH and control groups.

Gene	Genotype/allele	EH group *N* (%)	Control group *N* (%)	*x* ^2^	*P*	OR (95% CI)
rs4459609	CC	46	7	1.99	0.37	
	CA	201	50			
	AA	153	43			
	C	293	64	1.49	0.22	0.81 (0.59, 1.13)
	A	507	136			

rs4291	AA	167	41	0.03	0.99	
	AT	191	48			
	TT	42	11			
	A	525	130	0.03	0.87	0.97 (0.70, 1.34)
	T	275	70			

rs4309	CC	51	6	4.90	0.09	
	CT	178	54			
	TT	171	40			
	C	280	66	0.28	0.60	0.92 (0.66, 1.27)
	T	520	134			

rs4331	GG	177	41	2.74	0.25	
	GA	195	47			
	AA	28	12			
	G	549	129	1.25	0.26	0.83 (0.60, 1.15)
	A	251	71			

rs4343	GG	31	8	0.02	0.99	
	GA	191	47			
	AA	178	45			
	G	253	63	0.001	0.97	0.99 (0.71, 1.39)
	A	547	137			

rs4461142	CC	129	29	1.90	0.39	
	CT	213	51			
	TT	58	20			
	C	471	118	0.001	0.97	1.01 (0.73, 1.38)
	T	329	82			

ACE rs12709426	AA	400 (100)	100 (100)	0	1	

Note: ^∗^*P* < 0.05.

**Table 5:** The interaction of ACE gene polymorphism in the EH groups.

Gene	Training balanced accuracy	Testing balanced accuracy	CV consistency	*P*	OR (95% CI)
rs4291, rs4309, rs4461142^∗^	0.6294	0.6112	10/10	<0.01	3.52 (2.20, 5.63)
rs4291, rs4461142	0.6056	0.5900	10/10	<0.01	2.45 (1.56, 3.83)
rs4459609, rs12709426, rs4291, rs4309, rs4461142, rs4331	0.6517	0.5612	10/10	<0.01	4.07 (2.55, 6.48)

## Data Availability

Data used to support the findings of this study will be available upon reasonable request.
